# From Routine to Risk: Medical Liability and the Legal Implications of Cataract Surgery in the Age of Trivialization

**DOI:** 10.3390/jcm14196838

**Published:** 2025-09-26

**Authors:** Matteo Nioi, Pietro Emanuele Napoli, Domenico Nieddu, Alberto Chighine, Antonio Carai, Ernesto d’Aloja

**Affiliations:** 1 Forensic Medicine Unit, Department of Medical Sciences and Public Health, University of Cagliari, 09040 Cagliari, Sardinia, Italy; nioimatteo@gmail.com (M.N.); domenico.nieddu@gmail.com (D.N.); alberto.chighine@unica.it (A.C.); antonio.carai@unica.it (A.C.); ernestodaloja@gmail.com (E.d.); 2 Department of Surgical Sciences, Eye Clinic, University of Cagliari, 09124 Cagliari, Sardinia, Italy; 3 Studio Oculistico Dr. Pietro Emanuele Napoli, 09014 Carloforte, Sardinia, Italy

**Keywords:** cataract surgery, malpractice, Gelli-Bianco Law, informed consent, posterior capsule rupture, surgical complications, ophthalmic litigation, Optical Coherence Tomography, patient safety, intraoperative video recording

## Abstract

Cataract surgery is the most common eye operation worldwide and is regarded as one of the safest procedures in medicine. Yet, despite its low complication rates, it generates a disproportionate share of litigation. The gap between excellent safety profiles and rising medico-legal claims is driven less by surgical outcomes than by patient expectations, often shaped by healthcare marketing and the promise of risk-free recovery. This narrative review explores the clinical and legal dimensions of cataract surgery, focusing on complications, perioperative risk factors, and medico-legal concepts of predictability and preventability. Particular emphasis is given to European frameworks, with the Italian Gelli-Bianco Law (Law No. 24/2017) providing a model of accountability that balances innovation and patient safety. Analysis shows that liability exposure spans all phases of surgery: preoperative (inadequate consent, poor documentation), intraoperative (posterior capsule rupture, zonular instability), and postoperative (endophthalmitis, poor follow-up). Practical strategies for risk reduction include advanced imaging such as macular OCT, rigorous adherence to updated guidelines, systematic video recording, and transparent perioperative communication. Patient-reported outcomes further highlight that satisfaction depends more on visual quality and dialogue than on spectacle independence. By translating legal principles into clinical strategies, this review offers surgeons actionable “surgical–legal pearls” to improve outcomes, strengthen patient trust, and reduce medico-legal vulnerability in high-volume cataract surgery.

## 1. Introduction

Cataract surgery is among the most frequently performed procedures worldwide and remains the leading intervention to address reversible blindness. While it is routine in high-income countries, cataract continues to be a major cause of visual impairment in low- and middle-income regions due to restricted access to surgical care. In Europe, demographic aging has markedly increased demand for cataract surgery. In Italy, for example, the incidence rose from 657.59 per 100,000 individuals in 2011 to 916.43 in 2018, reflecting both technological advances and population shifts [1].

Cataract surgery is considered one of the safest and most effective procedures in modern medicine. Nonetheless, its portrayal in public communication often emphasizes simplicity and certainty of success. Marketing strategies in the private sector commonly highlight rapid recovery and advanced technology, which may inadvertently foster unrealistic patient expectations. Patients are thus led to believe that outcomes should invariably be excellent, regardless of individual ocular conditions, and that perfect visual acuity is the expected result. However, patient-reported outcomes show that satisfaction is not determined solely by visual acuity or spectacle independence, but is strongly influenced by subjective visual quality, the presence of symptoms such as dysphotopsia, and the quality of perioperative communication [2]. This gap between promotional narratives and clinical reality fosters dissatisfaction and may trigger medico-legal claims. Although complications are relatively uncommon, they may still result in litigation, and ophthalmologists are frequently named in such cases—even when outcomes are not attributable to clinical negligence. The increase in medico-legal claims is largely explained by demographic aging and the consequent rise in surgical volumes, as cataract extraction is the most frequently performed procedure in ophthalmology (≈3 million cases annually in the United States). Indeed, medico-legal exposure closely parallels procedural frequency, with up to 95% of ophthalmologists facing litigation at some point in their careers. Unrealistic expectations should therefore be regarded as a secondary, amplifying factor rather than the primary determinant of litigation [3,4,5,6].

### 1.1. Situation in Europe

Across Europe, medical liability frameworks display considerable heterogeneity. These differences stem from historical legal traditions, healthcare system structures, and cultural attitudes toward accountability and patient protection. Systems vary in their reliance on civil versus criminal law and in whether they adopt fault-based or no-fault compensation schemes. Nordic countries generally employ no-fault mechanisms that prioritize efficient compensation without assigning blame. France applies a hybrid model, combining fault-based liability with state-funded compensation for adverse events not attributable to negligence. Italy follows a dual-track system encompassing both criminal and civil liability, whereas Germany and most Central European jurisdictions predominantly adhere to fault-based regimes. In the Balkans and parts of Eastern Europe, several states have codified autonomous criminal offences of medical malpractice, distinguishing them from Western approaches. Overall, European medico-legal systems oscillate between accountability through negligence-based standards and systemic solidarity through no-fault or mixed compensation models.

#### 1.1.1. Criminal Law

Criminal liability for medical malpractice in Europe can be broadly categorized into three models.

**Figure 1:**
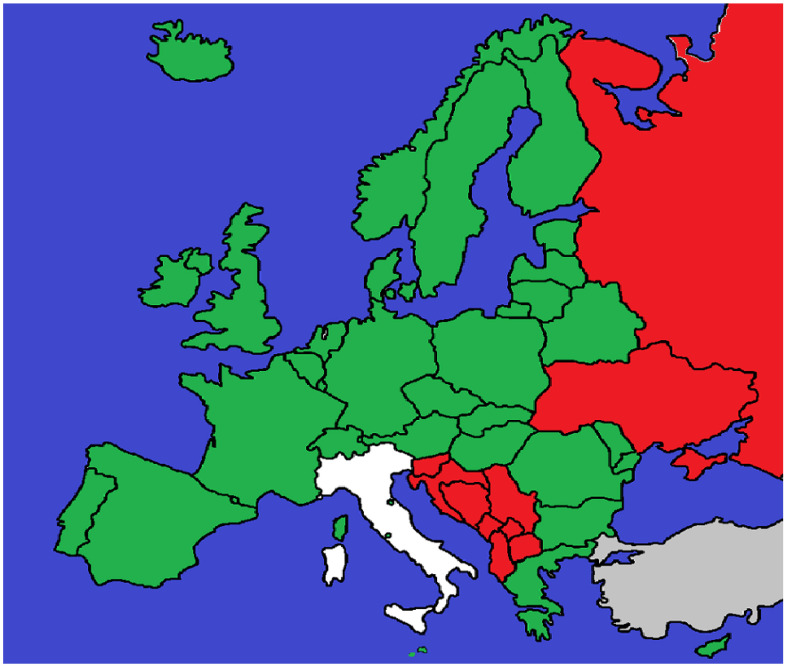
Countries shown in green prosecute medical malpractice under general criminal offences such as negligent bodily injury or negligent homicide. Jurisdictions in red have introduced an autonomous offence of medical malpractice into their penal codes. Italy is shown in white, as it applies general offences but provides a statutory defence under the Gelli-Bianco Law (Art. 590-sexies CC). Areas in grey indicate jurisdictions for which no reliable or accessible data could be identified.

The first and most widespread is the application of general criminal offences. Physicians are prosecuted under the same provisions as other citizens, typically negligent bodily injury, negligent homicide, or negligent manslaughter in common law systems. This model applies in Germany, France, the United Kingdom, Switzerland, Austria, Ireland, Spain, Portugal, Belgium, the Netherlands, Greece, Poland, Hungary, the Czech Republic, Slovakia, Romania, Bulgaria, the Nordic states, and microstates such as San Marino, Liechtenstein, Monaco, Andorra, and Vatican City. Its main strength lies in flexibility, but it often generates uncertainty in causation analysis and in defining the threshold for criminal negligence. The second model involves the creation of an autonomous offence of medical malpractice. This is characteristic of the former Yugoslav republics—Croatia, Serbia, Bosnia and Herzegovina, Montenegro, North Macedonia, Albania, and Kosovo—as well as Eastern jurisdictions such as Ukraine. These provisions explicitly criminalize negligent treatment by healthcare professionals (nesavjesno liječenje, nesavesno lečenje bolesnika). While intended to strengthen patient protection, they are often criticized for encouraging defensive medicine and resulting in relatively few convictions due to the difficulty of proving causation. Finally, Italy represents a sui generis model. While still applying the general offences of negligent homicide (Art. 589 CC) and bodily injury (Art. 590 CC), the 2017 “Gelli-Bianco Law” introduced Article 590-sexies CC, which exempts physicians from punishment when they act in compliance with accredited clinical guidelines or good practices (Figure 1). This statutory defence modifies the application of general offences, aiming to reduce over-criminalization and limit defensive medicine while maintaining accountability in cases of gross negligence (Appendix A).

#### 1.1.2. Civil Law

Civil liability for medical malpractice in Europe can be broadly categorized into three models: fault-based regimes, no-fault schemes, and hybrid systems. The fault-based approach remains predominant across most jurisdictions in Western, Central, and Eastern Europe. Under this model, patients must prove negligence, causation, and damages to obtain compensation. While this framework reinforces individual accountability and professional standards, it is frequently criticized for generating lengthy proceedings, uneven access to justice, and the persistence of defensive medical practices.

By contrast, the Nordic countries—Sweden, Finland, Norway, and Denmark—operate comprehensive no-fault compensation systems, financed through public resources. These mechanisms grant compensation without requiring proof of fault, prioritizing efficiency, rapid resolution, and equity while reducing the burden of litigation. Nonetheless, critics argue that no-fault systems may erode individual accountability and impose significant financial demands on healthcare budgets.

A hybrid model is exemplified by France, where the ONIAM (Office National d’Indemnisation des Accidents Médicaux) supplements traditional litigation by compensating adverse outcomes not attributable to negligence. This dual-track approach combines the deterrent effect of fault-based liability with a solidarity-based safety net for injuries arising from unavoidable risks (Appendix B).

**Figure 2:**
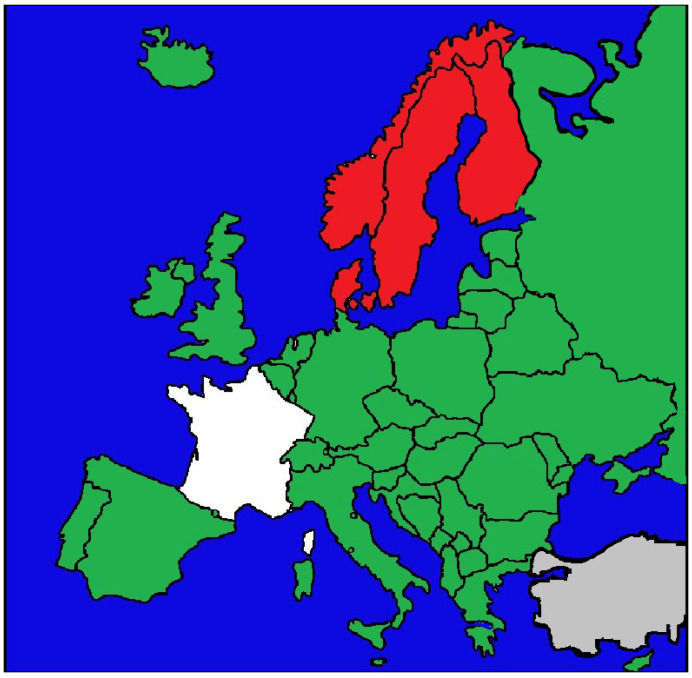
Countries shown in green apply a fault-based model of civil liability for medical malpractice, requiring patients to prove negligence, causation, and damages. The Nordic countries, shown in red, operate no-fault systems that provide compensation irrespective of negligence. France is shown in white, as it applies a hybrid model in which fault-based liability is supplemented by the state-funded ONIAM scheme, compensating adverse events not attributable to negligence. Areas in grey indicate jurisdictions for which no reliable or accessible data could be identified.

Overall, Europe presents a spectrum of civil liability frameworks (Figure 2). While fault-based systems remain the dominant paradigm, the expansion of no-fault and hybrid mechanisms illustrates a gradual shift in some jurisdictions toward prioritizing equitable access to compensation and systemic solidarity over the strict attribution of blame [7,8,9,10,11,12,13].

### 1.2. The Italian Framework

The Gelli-Bianco Law (Law No. 24/2017) reshaped Italy’s medical liability framework by introducing a dual-track system that clearly distinguishes between civil and criminal responsibility. Central to the law are the principles of *predictability* and *preventability*, which serve to differentiate medical errors from complications. An error is defined as an adverse event that is both foreseeable and avoidable, and therefore attributable to a deviation from accepted standards of care. By contrast, a complication is an adverse outcome that, although sometimes preventable, cannot always be predicted in advance, reflecting the intrinsic uncertainty of clinical practice.

Adherence to accredited clinical guidelines plays a pivotal role. In criminal proceedings, compliance with such guidelines may exempt physicians from punishment, limiting liability to cases of gross negligence. In civil proceedings, conformity to guidelines constitutes a cornerstone of the physician’s defense, shifting the burden of proof to the claimant once harm and causation have been established.

The law also reinforced informed consent and risk management as both legal and ethical cornerstones of clinical practice. Implementing decrees further operationalized these principles by mandating compulsory insurance for healthcare professionals and facilities, establishing minimum coverage thresholds, introducing claims-made policies with retroactive and run-off protection, and requiring the creation of dedicated risk and claims-reserve funds. Transparency obligations were also strengthened, with facilities required to publish data on compensated claims (Appendix C and Appendix D).

Together, these provisions aim to curb defensive medicine, enhance patient protection, and ensure financial sustainability while safeguarding professional accountability within the Italian healthcare system [12,13].

### 1.3. Assessment of Causation in Different Contexts and Jurisdictions

Demonstrating causation remains one of the most delicate aspects of medical liability and is often the decisive element in litigation. The fundamental requirement is to establish a causal link between professional conduct and patient harm, but evidentiary thresholds vary considerably across Europe. In criminal law, proof “beyond a reasonable doubt” is required—a stringent standard that frequently limits convictions in complex cases involving multiple contributing factors. Civil proceedings apply the lower standard of the “balance of probabilities,” which facilitates compensation but also perpetuates defensive practices in fault-based systems. In no-fault jurisdictions, such as the Nordic countries, causation is approached pragmatically: compensation is awarded when harm is plausibly connected to medical treatment, even in the absence of proven negligence. Hybrid systems, such as that of France, combine these logics, offering state-funded compensation for unavoidable adverse events alongside traditional litigation. Despite these jurisdictional differences, the medico-legal implications converge on a key point: clinical management must aim to minimize the occurrence and impact of complications. Whether liability is assessed under strict, probabilistic, or pragmatic standards, the prevention of avoidable harm remains the most effective safeguard for both patients and physicians. This requires adherence to scientific principles, meticulous perioperative planning, and the systematic adoption of practices designed to mitigate foreseeable risks. In this sense, the legal diversity across Europe underscores a common scientific imperative: complications may be judged differently in court, but they must be prevented and managed according to the same evidence-based standards. This perspective introduces the need to examine the specific phases of cataract surgery and their corresponding medico-legal vulnerabilities.

## 2. Materials and Methods

This narrative review was conducted to examine the medico-legal aspects of cataract surgery in Italy, with particular emphasis on the implications of the Gelli-Bianco Law and the associated risks of medical liability. A structured yet flexible methodology was adopted to identify relevant clinical and legal literature, analyze key complications and risk factors, and explore preventive strategies. A comprehensive search was performed across medical and legal databases—including PubMed, Scopus, Google Scholar, and ScienceDirect—focusing on English-language sources. Search terms combined “cataract surgery,” “medical liability,” “Gelli-Bianco Law,” “surgical complications,” “posterior capsule rupture,” and “medico-legal frameworks.” No date restrictions were applied, thereby capturing both historical developments and current practices. The review emphasized complications most frequently cited in malpractice claims, such as posterior capsule rupture, endophthalmitis, and intraocular lens dislocation. Relevant studies, case reports, and national and international clinical guidelines were examined to assess incidence, management strategies, and legal implications. Italian guidelines from the *Società Oftalmologica Italiana* (SOI) and the *Associazione Italiana Medici Oculisti* (AIMO) were analyzed alongside international best-practice recommendations [14,15,16,17]. On the legal side, particular attention was given to Law No. 24/2017 (Gelli-Bianco Law), which redefined civil and criminal liability in medical malpractice cases. Legal databases and scholarly commentaries were consulted to examine case law, jurisprudential trends, and interpretations concerning burden of proof, causality, and foreseeability in claims involving cataract surgery. Comparative analysis also considered European legal systems adopting no-fault or hybrid liability models. Preventive strategies were assessed, including adherence to clinical guidelines, thorough patient documentation, and comprehensive informed consent. Technological innovations—such as femtosecond laser-assisted cataract surgery, Scheimpflug imaging, and surgical video recording—were evaluated for their dual role in improving clinical outcomes and reducing medico-legal exposure. Finally, the review identified critical phases within the surgical pathway where both complication risk and legal vulnerability are heightened. These events were classified according to their predictability and preventability, underscoring how such factors shape clinical decision-making and legal accountability. This review integrates medical and legal perspectives to propose actionable recommendations that enhance patient safety while protecting healthcare professionals from unjust claims.

## 3. Results: Phases of Cataract Surgery and Their Medico-Legal Implications

Cataract surgery is a multi-phase process encompassing diagnosis, surgical indication, operative management, and postoperative care. Each stage presents distinct challenges with implications for both clinical outcomes and medico-legal liability. This section analyzes the most critical phases, highlighting areas of vulnerability and potential exposure to litigation.

### 3.1. Indication for Surgery

According to the guidelines of the *Società Oftalmologica Italiana* (SOI), the *Associazione Italiana Medici Oculisti* (AIMO), and the National Institute for Health and Care Excellence (NICE) [14,15,16,17], cataract surgery is primarily indicated to restore visual function in patients with lens opacity, to improve residual vision in the presence of comorbid ocular conditions, or to facilitate access to the posterior segment for diagnostic or therapeutic purposes [18,19,20]. Although visual acuity remains a central parameter for surgical indication, it is increasingly regarded as insufficient to capture the full extent of functional impairment or subjective complaints. Recent literature and clinical guidelines emphasize integrating quality-of-life metrics—such as patient-reported outcome measures (PROMs)—into surgical decision-making. Objective assessments, including Scheimpflug imaging and straylight measurement, have been suggested to refine surgical timing, although none are validated for routine use. Decision-making algorithms that combine visual acuity, PROMs, and preoperative data are emerging as promising tools to standardize indications [21,22,23,24].

The concept of functional vision—often defined as a visual acuity of 0.50 decimal (20/40 feet or 0.30 LogMAR)—is gaining recognition as both a clinical and legal threshold. Falling below this level may impair essential tasks such as driving. In Italy, a minimum binocular acuity of 1.00 decimal is required for private vehicle licenses, with at least 0.20 in the weaker eye. For professional drivers, the requirement rises to 1.40, underscoring the legal and practical importance of visual standards [25,26,27,28].

Beyond visual restoration, lens extraction has therapeutic relevance in certain conditions. The EAGLE randomized controlled trial demonstrated that early clear-lens extraction in selected patients with primary angle-closure disease (PAC/PACG) was more effective and cost-effective than laser peripheral iridotomy, thereby expanding cataract surgery indications to preventive and therapeutic settings in glaucoma management [29].

### 3.2. Pre-Surgery Evaluation and Medico-Legal Implications

The preoperative phase is critical for establishing clinical justification and minimizing legal risk. Surgeons must document both ocular and systemic conditions, including factors that could complicate surgery or limit postoperative outcomes. National and international guidelines emphasize excluding retinal pathologies that may compromise recovery [15,16,17]. Posterior capsule rupture (PCR)—a common intraoperative complication—is not invariably indicative of error, particularly in patients with pre-existing risk factors. In cases with limited expected benefit or advanced comorbidities, a detailed risk–benefit analysis should be explicitly recorded.

From a medico-legal perspective, documentation should also clarify the rationale for additional investigations. Advanced imaging—such as macular OCT—may be crucial in high-risk patients or in candidates for premium IOLs. The clinical and legal implications of OCT are addressed in Section 3.4. Structured frameworks such as the *Nationell Indikationsmodell för Katarakt Extraction* (NIKE), which integrates clinical indicators with patient-reported symptoms, can further refine patient selection and reduce liability [30]. Predictive tools combining anatomical and subjective data are also being developed to optimize outcomes and limit medico-legal exposure [18,19,22,23].

### 3.3. Informed Consent

Informed consent is a cornerstone of legal and ethical protection in cataract surgery. While oral consent was historically accepted in Italy, recent reforms have made written and signed consent mandatory, reflecting the growing emphasis on patient rights and accountability. Evidence shows that multimodal communication—including oral explanation, written material, and audiovisual tools—improves comprehension and recall of risks and options [31]. Consent should be procedure-specific, anticipate foreseeable intraoperative changes (e.g., conversion to extracapsular or intracapsular extraction), and remain revocable at any stage.

Beyond the signed document, best practice requires that the dialogue itself be documented in the medical record, as litigation often turns on what was communicated as much as on what was performed [17,31]. This is especially relevant in personalized surgery—such as premium IOL implantation or high-risk cases—where expectations are higher and costs may be borne directly by the patient. Recording the communication process provides proof of shared decision-making and protects against disputes arising from unmet expectations [17,32].

Informed consent should clearly state that no outcome can be guaranteed, while presenting a balanced account of risks and benefits. In Italy and other jurisdictions, best practice also entails having consent signed in the presence of a witness where required. Studies confirm that patients who perceive themselves as actively involved report higher satisfaction and are less inclined to litigate, regardless of the outcome [31,32,33]. Literature further indicates that the quality of communication and involvement—rather than exhaustive technical detail—is the key determinant of satisfaction and medico-legal protection [34,35].

### 3.4. Preoperative Examinations

Preoperative assessment must include systemic and ocular evaluations, as this phase is critical to reducing both surgical risk and medico-legal vulnerability. Systemic conditions such as diabetes mellitus and hypertension increase complication risk and should be explicitly documented [36,37]. National and international guidelines recommend a comprehensive history, targeted ocular imaging, and anesthetic risk assessment [6,7,19,26].

According to NICE NG77, optical biometry (or ultrasound if optical methods are not feasible) and keratometry are mandatory for all patients, while corneal topography is indicated in cases of irregular cornea, significant astigmatism, or a history of refractive surgery [17].

The role of macular OCT is debated. NICE recommends a selective approach, reserving OCT for patients with diabetes, unexplained visual loss, or suspected macular disease [17]. However, recent studies report that routine OCT screening uncovers occult macular pathology in ~13% of eyes otherwise eligible for surgery [38]. Broader OCT adoption, particularly in premium IOL candidates, has been advocated to minimize litigation risk. Documenting the rationale for either selective or universal OCT provides both clinical and legal protection.

Frameworks such as the NIKE model and predictive algorithms combining objective parameters with subjective data may further reduce unwarranted variability [20]. Integrating PROMs reflects a shift from visual acuity alone to a broader functional vision perspective [11,16].

Overall, rigorous preoperative assessment strengthens patient safety while providing essential legal defensibility.

### 3.5. The Surgical Procedure: Technical Complexity and Medico-Legal Relevance

Phacoemulsification is among the most frequently performed surgical procedures worldwide. Despite its high success rate, its technical intricacies demand surgical precision, meticulous documentation, and proactive risk management. From incision to IOL implantation, each step entails clinical judgment and medico-legal implications.

#### 3.5.1. Documentation and Risk Management

Intraoperative documentation is not merely administrative but a core safeguard in medico-legal defense. Operative notes should detail incision size, nucleus management, IOL choice and placement, and any deviation from protocol. Analyses of malpractice claims show that litigation often arises from poor documentation, even when outcomes are acceptable [39]. Prospective audits of high-volume centers reveal that nearly 20% of cases involve deviations from routine, with 5% classified as near misses [40]. Recording such events demonstrates transparency, strengthens safety, and enhances legal defensibility.

#### 3.5.2. Posterior Capsule Rupture (PCR) and Prevention

PCR remains the most feared intraoperative complication, with incidence ranging from 0.5% to 5% depending on complexity and surgeon experience [41,42]. Consequences include vitreous loss, IOL instability, cystoid macular edema, and retinal detachment [43]. Risk factors include posterior polar cataract, pseudoexfoliation, zonular weakness, small pupils, prior vitrectomy, and extremes of chamber depth [42]. Prevention requires refined planning: appropriate capsulorhexis size, preference for hydrodelineation in posterior polar cataracts, careful ultrasound modulation, and advanced phaco strategies (phaco-chop, stop-and-chop) [41,42,44]. Adjunctive technologies such as FLACS may enhance safety in selected cases [45]. When PCR occurs, standardized management (viscoelastic tamponade, anterior or pars plana vitrectomy, secondary IOL strategies) mitigates complications [41,42,43,44].

#### 3.5.3. Corneal Incisions and Endothelial Safety

Corneal incision design influences refractive and safety outcomes. Micro-incisions (1.8–2.2 mm) reduce induced astigmatism and edema compared to 3.0 mm incisions [46,47], particularly in patients with endothelial compromise. When combined with dispersive viscoelastics and meticulous wound construction, they optimize safety and recovery.

#### 3.5.4. Retained Lens Fragments and Vitreous Complications

Residual lens fragments can cause inflammation, hypertension, macular edema, or retinal detachment [44]. Incidence ranges 0.1–1% [46]. Anterior fragments often require early removal; posterior dislocation mandates careful vitrectomy and referral to vitreoretinal surgery [44,47]. Prevention includes cortical clean-up, capsule polishing, and use of modern phaco technologies [48].

#### 3.5.5. IOL Placement and Biometric Precision

Accurate IOL positioning underpins refractive success. Malposition or tilt can cause aberrations and dissatisfaction [49]. Swept-source OCT and intraoperative aberrometry improve accuracy [50]. Stability is influenced by ACD, AL, and LT. Capsulorhexis-fixated designs (e.g., FEMTIS) improve outcomes. When capsule support is insufficient, options include AC IOLs, scleral-fixated PC IOLs, and iris-fixated IOLs, each with risks [51,52].

#### 3.5.6. Endothelial Protection and FLACS

Endothelial integrity is vital, particularly in patients with diabetes, Fuchs dystrophy, or trauma. Strategies include dispersive viscoelastics, low-energy phaco, and reduced chamber turbulence [41,42,43]. FLACS reduces ultrasound energy and may preserve endothelium, especially in high-risk eyes [45,53,54].

#### 3.5.7. Posterior Capsular Opacification (PCO)

PCO is the leading late complication, due to lens epithelial cell proliferation [55,56]. Prevention involves cortical aspiration, capsule polishing, and sharp-edged IOLs [57]. Nd:YAG capsulotomy treats PCO but carries risks of IOL displacement, CME, or retinal detachment [58], risks often underdisclosed to patients.

#### 3.5.8. Suprachoroidal Hemorrhage and Intraoperative Emergencies

SCH is a rare but catastrophic complication (~0.03%) [59]. Risk factors include age, hypertension, vascular fragility, and anticoagulation. Signs include chamber shallowing and IOP rise [59,60]. Management requires rapid wound closure, IOP elevation, systemic stabilization, and repositioning. Patients with glaucoma are particularly vulnerable [61].

#### 3.5.9. Personalization and Shared Decision-Making

Personalized cataract surgery—through IOL selection, surgical technique, and perioperative planning—has growing ethical and legal weight, especially with premium IOLs. Shared decision-making improves satisfaction, aligns expectations, and reduces litigation [35,62]. Surgeons must document not only choices but the communication process, including risk disclosure and lack of guarantees. This documentation often distinguishes informed complications from inadequate disclosure.

#### 3.5.10. Surgical Safety Checklists and IOL Verification

Structured checklists reduce preventable errors. Wrong IOL implantation remains a frequent litigation cause and is a “never event” in the NHS. Since 2010, the Royal College of Ophthalmologists has promoted cataract-specific checklists, with >85% adoption [63]. WHO checklists reduce mortality and major complications across surgery, but compliance varies. Beyond technical safety, checklists strengthen communication and teamwork [64]. IOL verification and documented compliance also serve as medico-legal safeguards [65] (Table 1).

### 3.6. Postoperative Care: Adherence, Complications, and Legal Implications

#### 3.6.1. Compliance and Early Inflammatory Events

Successful postoperative outcomes depend on adherence to topical therapy, hygiene measures, and scheduled follow-up. Non-compliance—particularly in elderly or cognitively impaired patients—may precipitate anterior chamber inflammation, intraocular pressure (IOP) spikes, and secondary infection. Early inflammatory reactions are relatively common (≈8% after cataract surgery), typically resolving with corticosteroids and rarely leading to long-term sequelae [63]. Randomized evidence indicates that NSAID monotherapy is non-inferior to combination regimens, whereas depot “dropless” strategies may be less reliable [64]. From a medico-legal perspective, documenting both the prescribed regimen and the patient’s understanding of its importance is essential to demonstrate adherence to the standard of care.

#### 3.6.2. Infectious Endophthalmitis

Although infrequent—reported between 0.02% and 0.3%—infectious endophthalmitis remains one of the most catastrophic complications of cataract surgery [65,66,67]. Recent registry data spanning > 8.5 million U.S. procedures found an incidence of 0.04%, with higher rates when cataract surgery was combined with other intraocular procedures or when anterior vitrectomy was required [66]. A registry study reported a lower infection rate associated with immediate sequential bilateral cataract surgery (ISBCS) [67], whereas multicenter studies confirmed posterior capsule rupture and diabetes as independent risk factors for postoperative endophthalmitis [68]. Visual prognosis is often guarded, with many patients remaining below legal driving vision, although a subset may recover 20/40 or better [66]. While the classic indication for early pars plana vitrectomy was limited to light-perception vision, more recent series suggest functional benefit in selected cases beyond this threshold [69,70]. Prevention is paramount. Universal measures include povidone–iodine antisepsis, sterile draping, and meticulous wound construction. Intracameral antibiotics (cefuroxime or moxifloxacin) significantly reduce incidence and are now regarded as standard of care [68]. By contrast, reliance on topical antibiotics alone, inadequately hydrated or unsutured clear-corneal incisions, and poor compliance with perioperative hygiene protocols have been associated with higher rates and should be avoided [66,68].

Medico-legally, strict adherence to evidence-based prophylaxis—and clear documentation thereof—is decisive. Failure to implement recommended measures or to recognize early symptoms during follow-up may be interpreted as a breach of duty and frequently underpins litigation [68,69,70].

#### 3.6.3. Delayed Mechanical Complications

Beyond the early postoperative period, the most common mechanical complications are posterior capsular opacification (PCO) and late intraocular lens (IOL) dislocation. PCO remains the leading long-term cause of visual decline and often requires Nd:YAG capsulotomy. Late IOL dislocation, although less frequent, is a serious event that can result in irreversible vision loss if not promptly managed. Predisposing factors include pseudoexfoliation, prior vitrectomy, zonular weakness, and chronic intraocular inflammation [71,72]. Large registry analyses show that systemic comorbidities—diabetes, hypertension, chronic kidney disease, autoimmune disorders—significantly elevate the risk of severe postoperative complications, including IOL instability [71]. In uveitic eyes, both early and late complications are prevalent; PCO affects over half of cases and is frequently accompanied by cystoid macular edema, epiretinal membranes, and recurrent inflammation, all of which can exacerbate IOL instability [72]. Management typically entails surgical repositioning or IOL exchange, each carrying additional risk. When baseline risk factors (e.g., pseudoexfoliation or uveitis) are overlooked—or inadequately discussed during informed consent—litigation risk increases. Thorough preoperative identification of systemic and ocular risk, coupled with precise documentation of patient counseling, is therefore the cornerstone of clinical safety and medico-legal defensibility.

#### 3.6.4. Legal and Ethical Relevance

Postoperative complications—especially infectious endophthalmitis and late IOL dislocation—are among the most frequent triggers of medico-legal disputes. Legal evaluations typically ask whether the complication was foreseeable, whether evidence-based preventive protocols were implemented, and whether documentation demonstrates continuity of care [54,55]. Omissions in prophylaxis, delayed recognition, or inadequate referral may constitute breaches of duty, particularly when they result in avoidable harm.

Ethical responsibilities are equally central. Surgeons must maintain transparent communication not only at consent but throughout follow-up: disclose risks, promptly report adverse events, and clearly explain management options. Equity requires facilitating access to follow-up and accommodating vulnerable populations (e.g., elderly or visually impaired patients), avoiding paternalistic minimization of risk [73,74].

Timely recognition, appropriate referral, and detailed documentation thus serve a dual role: they represent best clinical practice and critical medico-legal safeguards, while fulfilling the ethical duty to act in the patient’s best interests, preserve trust, and ensure transparency across the care pathway (Table 2 and Table 3).

## 4. Discussion

Cataract surgery, the most widely performed ophthalmic procedure worldwide, embodies a paradox: it combines extremely low complication rates with disproportionately high medico-legal exposure. Its portrayal in public discourse—often fueled by marketing, social media, and anecdotal testimonials presenting it as a rapid, risk-free route to “perfect vision”—creates unrealistic expectations that, when unmet, frequently result in litigation [74,76,77]. Large-scale analyses confirm that posterior capsule rupture (PCR), retinal detachment (RD), and endophthalmitis, although rare (0.1–0.2%), remain the leading complications cited in malpractice claims [74,76,77]. The perceived unacceptability of such adverse outcomes in what is considered a “routine” operation amplifies their medico-legal significance. Geographical comparisons underscore systemic differences. In Korea, 42% of cases ended in plaintiff verdicts, whereas in the U.S. and U.K. most verdicts favored the defense [77,78,79]. Indemnity amounts, however, were markedly higher in the U.S., frequently exceeding one million dollars, compared with substantially lower awards in Asia and Europe. These discrepancies reflect not only differences in malpractice law and insurance systems but also cultural expectations regarding patients’ “right” to flawless results.

Across jurisdictions, informed consent consistently emerges as the central medico-legal battleground. Historical analyses identified incomplete consent as fertile ground for disputes [79], and contemporary data confirm its pivotal role: inadequate counseling explains nearly half of successful claims in Korea [78] and about 10% in the U.S. [77]. Patients often contend that they were not informed of residual ametropia, dysphotopsia, or the possibility of requiring spectacles despite premium IOL implantation. This shift from a restorative to a refractive paradigm means that even clinically successful outcomes may be perceived as failures when expectations are unmet. Effective consent must therefore be understood as a documented process of dialogue and shared decision-making, not a mere signature.

Documentation and perioperative management are equally decisive. Analyses of closed claims reveal that poor or delayed operative notes, defensive records, and inadequate follow-up frequently render cases indefensible, irrespective of the underlying complication [79]. Errors in biometry or IOL power selection, although uncommon, often result in liability [79]. These findings highlight that medico-legal risk extends beyond technical proficiency to organizational safeguards: accurate diagnostics, robust checklists, timely complication recognition, and transparent documentation represent critical protective measures.

Historical perspectives confirm the persistence of these issues. Failures in communication, refractive errors, and delayed diagnosis of infection have driven litigation across both the 20th and 21st centuries [79,80], despite significant technological progress. Innovation reduces technical risk but cannot eliminate disputes rooted in human and relational factors. Ultimately, reducing medico-legal vulnerability requires not only surgical excellence but also a sustained culture of safety, communication, and expectation management.

In summary, cataract surgery exemplifies how a highly effective and standardized procedure remains a prominent source of litigation due to its ubiquity, its frequent portrayal as risk-free, and the growing refractive ambitions of patients and providers. Medico-legal lessons converge across contexts: rare but severe complications dominate claims; informed consent is decisive; documentation and timely management are critical; and societal perceptions, amplified by media, often transform dissatisfaction into legal action. Addressing these dimensions requires aligning clinical reality with patient expectations through ethical communication, personalized planning, and transparent documentation—strategies that safeguard both patients and surgeons.

### Future Perspectives

Looking ahead, several strategies may strengthen both surgical safety and medico-legal resilience. Transparent communication is essential to bridge the gap between patient expectations and clinical reality, counteracting the widespread perception of cataract surgery as inherently risk-free. Comprehensive training and supervised experience remain fundamental for young surgeons, while simulation-based education provides valuable preparation prior to independent practice. Digitalized medical records and advanced diagnostics such as Scheimpflug imaging and straylight analysis can further enhance surgical planning while providing defensible evidence in litigation.

Equally important is strict adherence to updated clinical guidelines, which must increasingly incorporate innovations such as femtosecond laser-assisted cataract surgery and intraoperative OCT. Routine video recording of procedures, once optional, is likely to become a standard safeguard, offering objective evidence in case of dispute. Finally, international harmonization of objective criteria for surgical indication, complication reporting, and postoperative follow-up—together with the integration of real-time imaging and AI-based decision-support tools—may reduce variability, improve transparency, and foster both safer clinical practice and stronger medico-legal protection (Table 4).

## 5. Conclusions

Cataract surgery represents a paradigm of modern ophthalmology: a high-volume, highly successful procedure that nonetheless carries the potential for complications leading to litigation. The growing intersection of surgical complexity, elevated patient expectations, and medico-legal accountability demands more than technical mastery—it requires strategic foresight. Excellence in cataract surgery today is inseparable from effective risk management. This begins in the preoperative phase, where accurate documentation, structured risk stratification, and tailored informed consent form the foundation not only for optimal clinical outcomes but also for legal resilience. Clinical guidelines, decision-support tools, and structured communication are therefore pivotal.

Intraoperatively, advanced technologies—such as femtosecond lasers, intraoperative OCT, and refined biometric planning—enhance safety and precision. Their true medico-legal value, however, lies in integration with meticulous reporting and documentation. While such measures cannot eliminate liability, they significantly strengthen the surgeon’s position when adverse events occur. Postoperative follow-up, too often underestimated, emerges as a critical phase in which lapses in monitoring, communication, or documentation may determine the legal outcome, even when complications are inherently unpredictable.

The Italian Gelli-Bianco Law underscores a principle increasingly recognized across European systems: complications do not equate to malpractice, and liability must be contextualized within the framework of clinical complexity and adherence to evidence-based standards. Properly implemented, this model can promote a safer and more equitable medico-legal environment for both patients and practitioners.

Ultimately, the evolution of cataract surgery must extend beyond technological innovation to encompass cultural transformation—embracing transparency, rigorous documentation, and genuine shared decision-making as indispensable components of clinical excellence and sustainable medico-legal practice.

## Figures and Tables

**Table 1:** Surgical Pearls.

Area	Surgical Pearl	Rationale (Clinical and Medico-Legal)	References
**Posterior Capsule Rupture (PCR)**	Place a second instrument beneath the phaco tip during quadrant removal or hydrodissection.	Provides posterior capsule support and reduces the risk of rupture. Demonstrates surgical diligence and adherence to best practice, which is relevant in medico-legal evaluations.	Agarwal 2013 [41]; Upasani 2024 [48]
**Cortex removal/I&A**	Use angled or bimanual I/A tips to access subincisional cortex.	Facilitates safe removal of peripheral cortex, minimizes capsular traction and tears. Prevents foreseeable complications, strengthening medico-legal defensibility.	Buratto 2013 [44]; Upasani 2024 [48]
**Iris prolapse/IFIS**	Prepare Malyugin ring or iris hooks in at-risk patients; adjust fluidics by reducing flow and pressure during hydrodissection.	Stabilizes the iris, prevents prolapse, and reduces surgical time. Anticipating and managing IFIS reflects adherence to recognized standards of care.	Liu & Bardan 2021 [65]
**Corneal endothelium & wound safety**	Use dispersive OVDs, low ultrasound power, and microincisions (1.8–2.2 mm). Ensure meticulous wound construction with stromal hydration or sutures if needed.	Protects the corneal endothelium, reduces postoperative edema, and prevents wound leaks. Documentation of such measures supports legal defense in case of postoperative complications.	Chen 2021 [45]; Abell 2015 [53]; Conrad-Hengerer 2013 [54].
**Wound construction & Endophthalmitis prevention**	Apply povidone–iodine antisepsis, sterile draping, and administer intracameral antibiotics (cefuroxime or moxifloxacin).	Minimizes bacterial ingress and reduces endophthalmitis risk. Omission or inadequate documentation may be judged as a breach of duty.	Pershing 2020 [66]; Friling 2022 [67]; Saba 2024 [68]; Soliman 2021 [69]; Lanza 2021 [70].
**IOL verification & Surgical time-out**	Implement dual IOL verification: surgeon and nurse pre-implant, and full team confirmation post-draping.	Prevents ‘never events’ such as wrong IOL or wrong eye. Signed checklists provide strong medico-legal evidence of compliance with safety protocols.	WHO Checklist; Royal College of Ophthalmologists 2010 [64]
**Postoperative compliance**	Initiate topical therapy three days before surgery; consider intracameral antibiotics intraoperatively; adopt simplified postoperative regimens (e.g., NSAID monotherapy).	Improves adherence and reduces early postoperative inflammation. Therapy started under surgeon supervision is easier to document for medico-legal defense.	Mohammadpour 2007 [63]

PCR = Posterior Capsule Rupture; I/A = Irrigation & Aspiration; IFIS = Intraoperative Floppy Iris Syndrome; IOL = Intraocular Lens; OVD = Ophthalmic Viscosurgical Device. These pearls summarize practical measures that enhance surgical safety, improve clinical outcomes, and provide documented adherence to standards of care with direct medico-legal relevance.

**Table 2:** Key Considerations in Cataract Surgery: Clinical and Medico-Legal Aspects.

Category	Details
**Primary Indications for Surgery**	Improve visual function in patients with cataracts, even in the presence of other ocular diseases [15,16,17]. Maximize remaining vision in patients with comorbidities [16]. Facilitate examination of the posterior segment and prevent disease progression [17,18,19,20].
**Additional Factors for Surgery**	Visual acuity loss due to lens opacity remains a key driver for surgery [15,16,17]. Impact on daily functioning and quality of life must be considered [21,22,23,24].
**Objective Measurement Systems**	Devices such as Scheimpflug imaging and straylight measurements support surgical timing decisions but are not yet validated for routine clinical use [17,18,19,20,21].
**Pre-Surgery Documentation**	Thorough records must detail the patient’s clinical condition and any comorbidities that could affect surgery outcomes (e.g., glaucoma, diabetic retinopathy) [15,16,17,26].
**Medico-Legal Considerations**	Alternative diagnoses and comorbidities should be assessed and documented to limit liability [15,16,17]. Decision tools and PROMs (patient-reported outcome measures) can help balance risk–benefit discussions [22].
**Informed Consent**	Must be specific, revocable, and provided in multiple formats (oral, written, visual) to ensure understanding of risks, benefits, and alternatives [31,32,33,34,35].

This table summarizes the main clinical and legal considerations in the preoperative phase of cataract surgery. It includes criteria for surgical indication, the role of objective measurement systems, documentation requirements, legal risk factors, and essential components of informed consent. The integration of these elements supports safe surgical planning and mitigates medico-legal exposure.

**Table 3:** Predictability and Preventability of Adverse Events in Cataract Surgery.

Phase	Complication	Predictable	Preventable	Notes
**Pre-operative**	Pre-existing ocular conditions	Yes	No	Diseases such as advanced diabetic retinopathy may complicate outcomes but are often unavoidable [4,45].
	Inadequate pupillary dilation	Yes	No	Despite mydriatic treatment, some patients show insufficient dilation, increasing surgical difficulty [10,27].
	Zonular weakness	Yes	Partially	Often linked to pseudoexfoliation; capsular tension rings may reduce, but not eliminate, risk [28,38].
	Low endothelial cell count	Yes	Partially	Scheimpflug imaging helps predict risk; endothelial protection measures reduce damage but don’t fully prevent it [31,41,57].
**Intra-operative**	Posterior capsule rupture (PCR)	Yes	Partially	Risk increases with dense cataracts; minimized by surgical expertise and careful phacoemulsification [36,38].
	Zonular dehiscence	Yes	Partially	Anticipated in pseudoexfoliation; prevented through careful handling and ring use [28,38].
	Vitreous prolapse	Yes	Partially	Occurs with PCR; good surgical response limits damage [36].
	Suprachoroidal hemorrhage	No	No	Rare and catastrophic; often unpredictable [45,48].
	Corneal edema	Yes	Yes	Preventable by using dispersive OVDs and low ultrasound power [40,75].
	Lens dislocation	Yes	Partially	Weak zonules or trauma are risk factors; preventive support rings help but may not avoid it entirely [38,56].
**Post-operative**	Endophthalmitis	No	Yes	Preventable through povidone–iodine prep, intracameral antibiotics, and early intervention [49,53,54].
	Posterior capsular opacification (PCO)	Yes	Partially	IOL design may delay it, but Nd:YAG capsulotomy is often needed [42,43].
	Increased intraocular pressure (IOP)	Yes	Yes	Common in glaucoma; managed with medications and early follow-up [51].
	In-the-bag IOL dislocation	No	No	Linked to pseudoexfoliation and prior vitrectomy; difficult to anticipate [57].

This table summarizes key complications across the pre-operative, intra-operative, and post-operative phases, evaluating their predictability and potential for prevention. Notes include relevant clinical references to assist with patient risk stratification, informed consent, and surgical planning.

**Table 4:** Proposed Solutions and Future Perspectives in Cataract Surgery.

Category	Proposed Solution	Future Perspective
**Transparent Communication**	Clear communication with patients regarding expectations, marketing claims, and surgical outcomes.	Encourage open discussions about surgical risks and realistic outcomes to manage expectations.
**Proper Training**	Ensure surgeons are adequately trained and experienced before performing procedures independently.	Continued investment in simulation training and supervised surgeries to build proficiency in young ophthalmologists.
**Clear Medical Records**	Maintain comprehensive documentation of pre-operative conditions, surgical plans, and outcomes.	Use of digital records with real-time updates to ensure completeness and accessibility during legal disputes.
**Thorough Pre-Surgery Assessments**	Perform detailed patient assessments to account for anatomical challenges or underlying conditions.	Adoption of advanced diagnostic tools (e.g., Scheimpflug imaging, straylight measurements) to optimize surgical timing.
**Adherence to Guidelines**	Follow established clinical guidelines unless clear deviations are justified and documented.	Regularly updated guidelines incorporating the latest technologies and research findings to align with modern practice.
**Routine Video Recording**	Record surgical procedures to provide objective evidence in case of legal challenges.	Integration of automated video recording systems as a standard practice to enhance transparency and accountability.
**Objective Criteria for Surgery**	Use objective diagnostic tools to determine the optimal timing for surgery (e.g., Scheimpflug imaging, straylight).	Standardization of these tools across clinical settings nationally and internationally, to reduce variability in decision-making.
**Technology Integration**	Implement technology like femtosecond laser and intraoperative imaging systems to improve surgical precision.	Further development of personalized surgical approaches with real-time imaging guidance to minimize complications.

The table highlights practical solutions and future perspectives designed to enhance patient safety and surgical precision while strengthening medico-legal defensibility. It emphasizes the central role of transparent communication, structured training, comprehensive documentation, adherence to updated guidelines, and the integration of emerging technologies. Abbreviations: OCT = Optical Coherence Tomography.

## Abbreviations

The following abbreviations are used in this manuscript:

PCR	Posterior Capsule Rupture
AI	Artificial Intelligence
ECCE	ExtraCapsular Cataract Extraction
EHR	Electronic Health Record
FLACS	Femtosecond Laser-Assisted Cataract Surgery
GBL	Gelli-Bianco Law
GDPR	General Data Protection Regulation
ICCE	IntraCapsular Cataract Extraction
IOL	Intraocular Lens
NIKE	Nationell Indikationsmodell för Katarakt Extraction
NHS	National Health System
OCT	Optical Coherence Tomography

## Appendix A

**Table A1:** Criminal liability for medical malpractice in Europe.

Category	Countries	Characteristics
**General offences (negligent bodily injury/negligent homicide)**	Germany, France, United Kingdom, Switzerland, Austria, Ireland, Portugal, Spain, Belgium, Netherlands, Greece, Poland, Hungary, Czech Republic, Slovakia, Romania, Bulgaria, Sweden, Finland, Norway, Denmark, Lithuania, Latvia, Estonia, Cyprus, Malta, San Marino, Vatican City, Liechtenstein, Andorra, Monaco, Luxembourg	Physicians prosecuted under ordinary negligence offences; no specific malpractice crime. Flexible but often uncertain in terms of causation and threshold of fault.
**Autonomous offence of medical malpractice**	Croatia, Serbia, Slovenia, Montenegro, Bosnia and Herzegovina, North Macedonia, Albania, Kosovo, Ukraine, Russia	Criminal codes explicitly include a separate offence of negligent medical treatment (nesavjesno liječenje, nesavesno lečenje bolesnika, etc.). Intended to enhance patient protection but criticized for over-criminalization, encouraging defensive medicine, and producing few convictions.
**General offences with statutory defence**	Italy	Applies ordinary offences of negligent homicide (Art. 589 CC) and negligent bodily injury (Art. 590 CC). But since the 2017 Gelli-Bianco Law, Article 590-sexies CC excludes punishment if the physician acted in accordance with accredited guidelines or good practices. A sui generis adjustment within the general model.

This table summarizes the main models of criminal liability applied to medical malpractice across European jurisdictions. Most countries prosecute physicians under general offences such as negligent bodily injury or negligent homicide, without a specific offence of medical malpractice. In contrast, several Balkan and Eastern European states have codified autonomous offences of negligent medical treatment, a choice intended to strengthen patient protection but often criticized for fostering defensive medicine and producing few convictions. Italy represents a sui generis model: while general offences still apply, Article 590-sexies of the Gelli-Bianco Law (2017) introduced a statutory defence that excludes punishment when physicians act in compliance with accredited guidelines or recognized good practices.

## Appendix B

**Table A2:** Civil liability for medical malpractice in Europe.

Category	Countries	Characteristics
**Fault-based systems**	Italy, Germany, Spain, Portugal, Switzerland, United Kingdom, Ireland, Belgium, Netherlands, Greece, Poland, Hungary, Czech Republic, Slovakia, Romania, Bulgaria, Austria, Luxembourg, Cyprus, Malta, San Marino, Vatican City, Liechtenstein, Andorra, Monaco, Croatia, Serbia, Slovenia, Montenegro, Bosnia and Herzegovina, North Macedonia, Albania, Kosovo, Russia, Ukraine	Patients must prove negligence, causation, and damages. Insurance plays a central role. Litigation is often lengthy and may encourage defensive medicine.
**No-fault systems**	Sweden, Finland, Norway, Denmark	Publicly funded schemes provide compensation regardless of negligence. Designed to ensure efficiency, reduce litigation, and strengthen patient protection, though they may reduce individual accountability.
**Hybrid/mixed systems**	France (ONIAM)	Combination of fault-based litigation and state-funded no-fault compensation for adverse events without negligence. Seeks to balance accountability with systemic solidarity.

This table summarizes the principal models of civil liability applied to medical malpractice across European jurisdictions. In most countries, fault-based systems prevail, requiring patients to prove negligence, causation, and damages, a process often criticized for lengthy litigation and the promotion of defensive medicine. By contrast, the Nordic countries operate no-fault schemes that provide compensation regardless of negligence, emphasizing efficiency and equity, though sometimes at the expense of individual accountability. France exemplifies a hybrid model, where fault-based litigation is complemented by the state-funded ONIAM scheme, designed to compensate adverse events not attributable to negligence, thereby balancing accountability with systemic solidarity.

## Appendix C

**Table A3:** Civil and Criminal Liability of Ophthalmologists under Italian Law.

Category	Domain	Burden of Proof	Fault	Responsibility type	Manager
**Independent** **practitioner**	Civil	Practitioner	Negligence, imprudence, incompetence.	Contractual	Not applicable
	Criminal	Prosecutor	Negligence, imprudence; incompetence excluded if guidelines are followed	Individual	Not applicable
**Employed practitioner**	Civil	Practitioner	Gross negligence if guidelines are complied with.	Contractual for the facility; extra-contractual for the practitioner	Not applicable
	Criminal	Prosecutor	Negligence, imprudence, not incompetence with guidelines.	Individual	Not applicable
**Healthcare facility**	Civil	Facility	Organizational Negligence (culpa in organizzando)	Contractual	Manager liable for organizational failures
	Criminal	Prosecutor	Gross negligence (culpa in vigilando/organizzando)	Not applicable	Manager liable under Legislative Decree D.Lgs 231/2001

This table outlines the legal responsibilities of ophthalmologists in Italy, distinguishing between independent and employed practitioners. In civil proceedings, independent professionals are contractually liable and, once harm and causation are established, must demonstrate adherence to recognized clinical standards. They may be held accountable for simple fault, including negligence, imprudence, and incompetence. In criminal proceedings, independent practitioners bear personal liability for negligence and imprudence, but not for incompetence, provided they have complied with accredited clinical guidelines. For employed practitioners, civil liability is generally extra-contractual and shared with the healthcare institution, unless a direct contractual agreement with the patient exists. In all criminal cases, the burden of proof rests with the public prosecutor.

## Appendix D

**Table A4:** Standards of Proof in Civil and Criminal Proceedings in Medical Liability Cases.

Domain	Percentage of Possibility Required	Legal Principle
**Civil law**	More likely than not (≥51%)	In dubio pro leso
**Criminal law**	Beyond a reasonable doubt (Near 100%)	In dubio pro reo

This table highlights the different thresholds of proof applied in civil and criminal proceedings involving medical liability. In civil cases, liability is established when it is more probable than not (≥51%) that the harm resulted from the defendant’s conduct, in accordance with the principle of in dubio pro leso, which favors the injured party in cases of uncertainty. By contrast, criminal proceedings require proof beyond a reasonable doubt (≈100%), consistent with the principle of in dubio pro reo, which safeguards the rights of the accused through the presumption of innocence.
